# Epidemiology of Drug Use and HIV-Related Risk Behaviors among People Who Inject Drugs in Mwanza, Tanzania

**DOI:** 10.1371/journal.pone.0145578

**Published:** 2015-12-23

**Authors:** Annabel Xulin Tan, Saidi Kapiga, Kaveh Khoshnood, R. Douglas Bruce

**Affiliations:** 1 Yale School of Public Health, New Haven, Connecticut, United States of America; 2 Mwanza Intervention Trials Unit, Mwanza, Tanzania; 3 Cornell Scott-Hill Health Center, New Haven, Connecticut, United States of America; 4 Yale University School of Medicine, New Haven, Connecticut, United States of America; Infectious Disease Service, UNITED STATES

## Abstract

Heroin trafficking and consumption has increased steadily over the past decade in Tanzania, but limited information regarding HIV and drug use exists for the city of Mwanza. Our study investigates the epidemiology of drug use, and HIV risk behaviors among drug users in the northwestern city of Mwanza. Using a combination of targeted sampling and participant referral, we recruited 480 participants in Mwanza between June and August 2014. The sample was 92% male. Seventy-nine (16.4%) participants reported injecting heroin, while 434 (90.4%) reported smoking heroin. Unstable housing and cohabitation status were the only socioeconomic characteristics significantly associated with heroin injection. More than half of heroin injectors left syringes in common locations, and half reported sharing needles and syringes. Other risk behaviors such as lack of condom use during sex, and the use of illicit drugs during sex was widely reported as well. Among the study sample, there was poor awareness of health risks posed by needle/syringe sharing and drug use. Our results show that heroin use and HIV risk related behaviors are pressing problems that should not be ignored in Mwanza. Harm reduction programs are urgently needed in this population.

## Introduction

The dual epidemic of HIV and substance use—specifically heroin injection—is a growing public health problem in Tanzania. It is well established that the injection of drugs is associated with HIV acquisition. Specifically, several studies conducted in Eastern Europe[1], China[2], South East Asia[3] and Russia[4] have demonstrated that injection drug use is a key driver for the spread of HIV infections, including in Tanzania.[5–7] Some people who inject drugs (PWID) engage in HIV risk-related behaviors, such as sharing syringes and other injection equipment, and having unprotected sex. [8–10] These behaviors are routes of HIV transmission within the PWID community and have the potential to spill over into the general population.[11]

In the past decade, the increase in the number of heroin users has fueled a rise in HIV infections among PWID in Tanzania.[12] While HIV prevalence in the country is estimated at 5.1%[13], HIV among injectors is fourfold the national rate: 28% among male injectors and 62% among female injectors.[14] The substantially higher HIV prevalence among females who inject drugs may be attributed to high risk sexual behaviors, including being involved in sex work. In Dar es Salaam, female heroin injectors are more likely to be living on the streets and to have multiple sex partners. [15] The high rates of HIV infections among PWID is likely to contribute in continued expansion of the HIV epidemic in Tanzania and is the impetus for the establishment of harm reduction services, such as methadone treatment, to reduce HIV transmissions in Dar es Salaam.

Limited data on heroin use in Tanzania and associated HIV risk factors is available mainly from Dar es Salaam and Zanzibar. Currently, 22 tons of heroin is trafficked through East Africa annually with 2.5 tons consumed locally. It is estimated that there are 30,000 PWID in mainland Tanzania. [16]. Anecdotal reports of heroin use along trucking routes from Dar es Salaam prompted an examination of heroin use in the far northwestern city of Mwanza, 1,139 km northwest of Dar es Salaam. In this study, we detail the epidemiology of substance use, heroin injection and its associated HIV-related risk behaviors in Mwanza, the second largest city in the nation. The objectives of this study are twofold. First, we want to describe the socioeconomic characteristics and epidemiology of substance use in a sample of people who use drugs in Mwanza, specifically comparing how PWID differ from non-PWID. Second, we want to highlight HIV-related knowledge and risk behaviors of the subgroup of PWID, in this sample, and determine if the risk behaviors are heterogeneous. To our knowledge, this is the first study of its kind to be conducted in Mwanza or elsewhere in Northwest Tanzania.

## Methods

### Fieldwork

From June to August 2014, 480 participants were recruited and interviewed about their substance use and associated risk behaviors. With this sample size, the study was estimated to have 90% statistical power to detect a difference of 0.04 using a two-sided binomial test with 95% confidence. Substance users were recruited based on a combination of targeted sampling and participant referral.[17] The sampling scheme was developed by AXT and SK using information collected by outreach workers who have extensive experience working with illicit substance users in Mwanza.

This information was then used to target certain neighborhoods in the city that were known as ‘hotspots.’ Neighborhood selection was then confirmed by direct observation of drug use by the interviewers. In total, 12 study areas were selected and 50–60 people were interviewed per area. Study areas were determined according to local administrative areas (wards) and then further divided according to street names where drug users were reported to congregate. We selected 7 wards, and out of these 7 wards, 12 streets. Data collection began at the street closest to the primary investigator’s office, and ended in the area furthest away.

Two outreach workers, who were trained by the investigators AXT and SK for a 5-day period prior to the start of data collection, conducted the interviews. Verbal consent was obtained for this study. Before conducting the interview, participants were read an information sheet in Swahili that contained all the details of the interview, and were informed of their right to not answer any question. The information sheet was also made available to the participants to read. Written consent was not obtained to protect the identities of the participants. Each participant was assigned a unique identification number and full confidentiality was maintained. Interviews were conducted individually in Swahili in various private, enclosed locations along the street where participants gathered.

The study and consent procedure was reviewed and approved by the institutional review boards for the protection of human subjects at Yale University, USA (HSC #1404013808) and at the National Institute of Medical Research (NIMR) in Tanzania in May 2014.

### Inclusion criteria

Eligible participants were those who self-reported to be above the age of 18, had injected drugs in the past 12 months or used any other kind of drug in the past 12 months. Many of those approached on the streets were preparing to use illicit drugs. Screening measures for drug use included self-report and visual inspection for injection marks on the arms, feet or legs. Illicit drug users were typically found in groups in alleyways, in rented rooms, abandoned homes, or common congregation areas such as football fields. Some participants also showed us their injection equipment and drugs voluntarily, as proof that they were drug injectors. Participants who completed the interview were provided a small meal, drink and/or snack after the interview. On average, each meal cost 958 TSH (USD$0.50).

Data were collected in Swahili using a structured questionnaire written by the investigators based on instruments employed by the World Health Organization (AUDIT survey), Tanzanian AIDS Prevention Program (MAT Pilot Program Client Assessment form) as well as previous instruments used in an alcohol study that have yielded promising results.[18] The questionnaire had 57 questions to collect information about demographic characteristics, non-injection and injection drug use, alcohol use and sexual risk behaviors. Responses were recorded on paper. The primary investigator provided feedback to the interviewers daily as part of the data editing and cleaning process. Feedback was based on 3 key points: completeness, accuracy, and consistency between answers. Open-ended questions were also reviewed closely to ensure that all responses were written legibly. After verification, written responses were then translated back into English.

### Statistical analyses and data management

Data were entered into an OpenClinica (Waltham, MA) database and analyzed with the statistical analysis software R (R Core Team, 2014). Data from all 480 participants were available for analysis. A key outcome of interest was heroin injection, defined as either yes or no. We fitted two logistic models to heroin injection use: socioeconomic characteristics and HIV risk related behaviors.

Predictors measured at continuous integer levels were recoded into discrete categories that reflected the distribution of data. Information collected via open-ended questions was coded and stratified as non-ordinal categories for use as independent variables. The reference category for each predictor was the one that had the lowest perceived HIV risk. We assessed correlations between each independent variable and the likelihood of injection. Predictors with the highest level of correlation and significance (p<0.2) were included in the multivariate models. Akaike Information Criterion (AIC) and deviance tests were used to compare models to one that included all independent significant variables associated with injection. The model with the lowest AIC value was used as the final reduced model. Incomplete data were omitted from the model.

To determine the best reduced multivariate model, we conducted factor analysis to find the strongest predictors for heroin injection. Factor analysis removes redundancy from a set of correlated variables, such that the final model is one that contains the most significant and highly correlated predictors.

Predictors were analyzed for independence, i.e., to examine potential significant interactions among the terms. All predictors were found to be independent. Interaction terms were not included in the final model as none were found to be significant.

Results are presented in the form of odds ratios with 95% confidence intervals. Significance of odds ratios was assessed using 95% confidence intervals and chi-squared tests. We used chi-squared tests to assess differences in socioeconomic characteristics and risk behaviors between PWID and non-PWID.

## Results

### Demographics

In our sample of 480 participants, we interviewed 442 males (92%) and 38 (8%) females. The overall mean age of our study sample was 28.8 years. The mean age among PWID was 29.0 years, while the mean age among those who had never injected drugs was 28.3 years. More than a tenth of the study population (10.4%) were living on the streets or considered themselves homeless, whereas170 (42.3%) rented rooms. A substantial fraction (69.4%) reported having a job as a primary source of income, where slightly less than a third (27.7%) reported stealing or pickpocketing as a primary source of income. Having a job, however, often included illicit means such as sex work or selling stolen goods.

### Epidemiology of drug use and HIV related risk behaviors

Among our study population, 65 (13.5%) reported currently injecting drugs. A majority of those who had a history of injection use remained injectors (82.3%). Almost everyone interviewed (99.4%) reported using drugs 7 days a week. Self-reported daily frequency of any kind of drug use ranged from once a day to a maximum of 25 times a day. The most commonly reported frequencies were: 3 times a day (20.8%), 5 times (20.0%), and 4 times (18.8%).

We found that many current injectors reported engaging in HIV-risk related injecting behaviors. For example, 44/65 (66.7%) reported sharing needles, 31/65 (47.7%) shared injection equipment and 41/65 (63.1%) left syringes in public locations. Although 58/65 (89.2%) of the current injectors reported cleaning injection equipment, only 32.7% cleaned their equipment with bleach. Most of the other PWID (43.1%) used potable water, a non-effective method of sterilization.[19]

We next explored the relationship between socioeconomic status and type of drug use. To assess socioeconomic status, we used the following measures: age, gender, education level, marital status, living arrangement and income source. However, we found that these factors were not significantly associated with heroin injection (summarized in Table 1).

**Table 1:** The associations between socioeconomic characteristics and history of ever injecting drugs among drug users in Mwanza, Tanzania.

	Ever* Heroin Injection (%) n = 79	Never Heroin Injection (%) n = 401	Unadjusted Odds ratio of Heroin Injection (95% CI)	p-value for independence**
**Age (+s.d.)**	29.0 ± 5.8	28.3 ± 6.4	0.98 (0.94–1.02)	0.52
**Age**				0.38
<20	4 (5.4)	19 (4.9)	1.51 (0.41–4.62)	
21–25	23 (31.1)	114 (29.3)	1.37 (0.70–2.75)	
26–30	21(28.4)	118 (30.4)	1.00	
31–35	18(24.3)	81 (20.9)	1.18 (0.55–2.52)	
>36	8 (10.8)	56 (14.4)	0.48 (0.13–1.36)	
**Gender**				0.14
Male	69 (87.3)	373 (93.0)	1.00	
Female	10 (12.7)	28 (7.0)	2.14 (0.91–4.59)	
**Education Level**				0.21
Never went to school	5 (6.3)	14 (3.5)	1.23 (0.27–5.38)	
Incomplete primary	9 (11.4)	73 (18.2)	0.50 (0.15–1.78)	
Completed primary	22 (27.8)	124 (30.9)	0.65 (0.23–2.11)	
Secondary (Form 1–4)	28(35.4)	143 (35.7)	0.71 (0.26–2.29)	
Secondary (Form 5–6)	7 (8.9)	27 (6.7)	1.19 (0.34–4.50)	
Post Secondary	8 (10.1)	20 (5.0)	1.00	
**Marital Status**				0.10
Single/never married	47 (64.6)	246 (61.3)	4.23 (1.49–17.8)	
Married or living as married (1 spouse)	7 (4.6)	65 (16.2)	1.00	
Married or living as married (>1 spouse)	2 (3.7)	11 (2.7)	4.61 (0.56–30.9)	
Separated/divorced	23 (27.7)	72 (18.0)	5.92 (1.91–26.02)	
Widowed	0 (0.0)	7 (1.7)	-	
**Housing Status**				0.22
Own house	5 (6.3)	32 (8.0)	1.00	
Rented house	7 (8.9)	19 (4.7)	3.50 (0.83–18.0)	
Renting room in guesthouse	6 (7.6)	12 (3.0)	5.83 (1.33–31.2)	
Renting room elsewhere	36 (45.6)	170 (42.3)	1.76 (0.58–7.65)	
Free room at friends or relative’s house	18 (22.7)	124 (30.9)	1.48 (0.46–6.62)	
On the Streets	7 (8.9)	43 (10.7)	1.90 (0.49–9.31)	
**Living with husband/wife**				0.19
Yes	9 (11.4)	72 (18.1)	1.00	
No	70 (88.6)	325 (81.9)	2.72 (1.16–7.99)	
**Income Source**				0.94
Family/friends	2 (2.5)	11 (2.8)	0.78 (0.44–1.39)	
Job	54 (68.4)	279 (69.6)	1.00	
Illicit activities	23 (29.1)	110 (27.4)	0.98 (0.14–3.99)	

* Ever injection was used as the primary variable, but it must be noted that the majority of the ever injectors were also current injectors (82%).

** Student’s t-test for continuous variables, chi-squared test for categorical variables.

A majority of PWID was in the 21–25 (31.1%) and 26–30 (28.4%) age groups. Similarly for non-PWID, a majority was in the 21–25 (29.3%) and 26–30 (30.4%) age groups. PWID included more females than non-PWID (12.7% vs. 7.0%, p>0.05). Fewer males were PWID than non-PWID (87.3% vs. 93.0%, p>0.05). More PWID had never gone to school compared to non-PWID (6.3% vs. 3.5%, p>0.05). A greater proportion of PWID reported some form of higher secondary education (8.9% vs. 6.7%, p>0.05) and post-secondary education compared to non-PWID (10.1% vs. 5.0%, p>0.05). More PWID lived without a spouse/partner compared to non-PWID (88.6% vs. 81.9%, p>0.05). Those who were separated or divorced were approximately 6 times (OR: 5.92; 95%: 1.91–26.02) more likely to inject heroin compared to those who are married with one spouse.

Room rental was the most common living arrangement among both PWID and non-PWID (45.6% vs. 42.3%, p>0.05). Living on the streets was slightly less prevalent among PWID than non-PWID (8.9% vs. 10.7%, p>0.05). Income sources differed very little between PWID and non-PWID (p>0.05). More than half of PWID and non-PWID have jobs as their main source of income (68.4% vs. 69.6%, p>0.05). Those who had jobs mostly reported that they sold household items, were drivers or engaged in sex work.

Table 2 shows a profile of the self-reported drug use among the study population. All injectors reported injecting heroin. This comprised (16.4%) of the entire sample. Other injected drugs included methamphetamine, cocaine and pharmaceutical drugs such as Diclopa and valium. Among the entire sample, the most common non-injected type of drug used was marijuana (95.0%) and heroin (90.4%). Heroin is commonly smoked with marijuana in joints called *kokteli* (translated to “cocktail”) and 86% of individuals reported smoking these. Less commonly reported among the study population was the combination of marijuana, non-injection heroin and pharmaceutical drugs (12.5%).

**Table 2:** Profile of self-reported drug use among drug users in Mwanza, Tanzania.

**Type of Drug Injected**	**N (%)**
Heroin	79/480 (16.4)
Methamphetamine	2/480 (0.4)
Cocaine	2/480 (0.4)
Pharmaceuticals	3/480 (0.6)
**Type of Drugs Not-injected**	
Marijuana	456/480 (95.0)
Non-injection heroin	434/480 (90.4)
Pharmaceutical	71/480 (14.8)
Hashish	13/480 (2.7)
Solvent	17/480 (3.5)
Marijuana and Non-Injection Heroin	413/480 (86.0)
Marijuana and Non-Injection Heroin and Pharmaceutical	60/480 (12.5)
**Combined Heroin Injection + Non-injected Drugs**	
Injection Heroin + Marijuana	61/79 (77.2)
Injection Heroin + Non-Injection Heroin	66/79 (83.5)
Injection Heroin + Non-injection Pharmaceuticals	20/79 (25.3)
Injection Heroin + Hashish	2/79 (2.5)
Injection Heroin + Solvent	10/79 (12.7)
Injection Heroin + Marijuana and non-injection heroin	50/79 (63.3)
Injection Heroin + Marijuana and Non-injection Heroin and Pharmaceutical	12/79 (15.2)

In addition to injecting heroin, smoking heroin was most commonly reported (83.0%) as a concurrent behavior, followed by smoking marijuana (77.2%). PWID also often engaged in smoking marijuana and heroin (63.3%). PWID also reported engaging in smoking heroin, marijuana and pharmaceutical drugs simultaneously (15.2%). These combinations of risky behaviors are not independent of each other.

### HIV-related risk behaviors

We show in Table 3 that PWIDs have used drugs for a longer period of time compared to non-PWIDs (8.73 years vs. 7.78 years, p>0.05). The range of drug use among PWID extends from 0–20 years. Among non-PWID, the range extends from 0–33 years. People who are more likely to engage in heroin injection are those who have been using drugs for a period of 11–15 years (29.2% vs. 18.8%, p>0.05) and 16–20 years (12.5% vs. 8.6%, p>0.05) compared to those who have been using for 0–5 years. There are significant associations between HIV risk behaviors and heroin injection include selling items for drugs, trading sex for drugs, and being arrested.

**Table 3:** The associations between risk behaviors and heroin injection among drug users in Mwanza, Tanzania.

	Ever Injection Drug Use	Never Injection Drug Use	Unadjusted Odds of Heroin Injection	p-value of independence
**Years since first use (+ s.d.)**	8.73 ± 5.1	7.78 ± 5.9	1.03 (0.98–1.07)	0.15
**Years since first use**				0.16
0–5 years	26 (36.1)	153 (41.1)	1.00	
6–10 years	16 (29.8)	111 (29.8)	0.93 (0.47–1.78)	
11–15 years	21 (29.2)	70 (18.8)	1.94 (1.02–3.63)	
16–20 years	9 (12.5)	32 (8.6)	1.81 (0.75–4.10)	
**Ever sold items for drugs**				<0.05
Yes	70 (88.6)	265 (66.3)	3.96 (2.02–8.73)	
No	9 (11.3)	135 (33.8)	1.00	
**Traded sex for drugs**				<0.05
Yes	29 (36.9)	56 (14.0)	3.55 (2.06–6.07)	
No	50 (63.0)	343 (85.9)	1.00	
**Arrested by law enforcers**				<0.05
Yes	77 (97.5)	333 (83.4)	7.75 (2.36–47.8)	
No	2 (2.5)	67 (16.8)	1.00	
**Spouse/partner is a drug user**				0.09
Yes	11 (13.9)	30 (7.5)	1.98 (0.92–4.06)	
No	66 (83.5)	363 (90.9)	1.00	
Don’t know	2 (2.5)	6 (1.5)	-	
**Use of drugs during sex**				0.18
Yes	71 (89.8)	333 (83.3)	1.78 (0.87–4.18)	
No	8 (10.1)	67 (16.8)	1.00	
**Main reason for starting drugs**				0.10
Peer pressure	63 (79.7)	320 (80.0)	-	
For fun	3 (3.8)	9 (2.3)	-	
Family problems	4 (5.1)	10 (2.5)	-	
Influence from family	3 (3.8)	17 (4.3)	-	
Other	4 (5.1)	11 (2.8)	-	
**Age of sexual debut**				0.84
11–13	27 (40.3)	150 (43.1)	1.19 (0.48–3.16)	
14–17	32 (47.8)	155 (44.5)	0.99 (0.48–2.41)	
18+	8 (11.9)	43 (12.3)	1.00	
**Number of sex partners in the past 30 days**				0.10
None	16 (20.2)	104 (27.1)	1.00	
1	25 (31.6)	135 (33.6)	1.20 (0.62–2.41)	
2–5	21 (26.6)	115 (28.7)	1.19 (0.59–2.43)	
>5	10 (12.7)	32 (7.9)	2.03 (0.81–4.87)	
**Use of condoms during vaginal sex**				0.08
None of the time	21 (26.6)	91 (22.8)	1.66 (0.86–3.21)	
Some of the time	29 (36.7)	98 (24.5)	2.13 (1.16–3.96)	
Most of the time	7 (8.9)	52 (13.0)	0.97 (0.37–2.31)	
All the time	5 (6.3)	52 (13.0)	1.00	
**Alcohol consumption**				0.37
Monthly or less	5 (10.9)	21 (9.5)	1.00	
2–4 times a month	9 (19.6)	53 (24.2)	0.71 (0.23–2.55)	
2–3 times a week	15 (32.6)	50 (22.8)	1.26 (0.43–4.27)	
4 or more times a week	17 (37.0)	95 (43.4)	0.75 (0.26–2.49)	
**Binge drinking frequency**				<0.05
Never	26 (56.5)	115 (52.5)	1.00	
Less than monthly	7 (15.2)	21 (9.6)	1.32 (0.48–3.29)	
Monthly	5 (10.9)	17 (7.8)	1.16 (0.36–3.24)	
Weekly	3 (6.5)	22 (10.0)	0.54 (0.12–1.70)	
Daily	2 (4.3)	44 (20.1)	0.18 (0.02–0.63)	

Peer pressure, defined as influence from members of his/her social circle, was one of the most commonly cited reasons for starting drug use among PWID and non-PWID (79.7% vs. 80.0%, p>0.05). Both PWID and non-PWID cited family problems as the reason for starting drugs (5.1% vs. 2.5%, p>0.05).

Risky sexual behaviors were found to be different between PWID and non-PWID, although these were not statistically significant. The proportion of both PWID and non-PWID that had their first sexual experience at a very young age was quite high: 43.1% and 40.3% respectively. More PWID than non-PWID reported having more than 5 sex partners in the past 30 days (12.7% vs. 7.9%, p>0.05). The use of condoms all of the time was less common among PWID than non-PWID (6.3% vs. 13.0%, p>0.05).

### HIV-related injection risk behaviors among injectors

We investigated HIV-related injection risk behaviors among injectors. A majority of those with a history of heroin injection are still current PWID (82.2%). Of the 65 current PWID, 67.7% have ever shared needles. Approximately half of PWID (47.7%) in our sample reported having shared injection equipment. A majority of the PWID (89.2%) reported cleaning their injection equipment and needles using soap (6.2%), alcohol (9.2%), bleach (29.2%), boiling water (4.6%), and cold water (38.5%).

The practice of flashblood was known among the study population, but rarely practiced. Flashblood is the practice that occurs when a PWID, after self-injecting heroin, draws blood back into the syringe. This blood is then injected by another PWID with the hope of having enough heroin in that blood sample to stave off withdrawal. [7] Overall, there were 6 people in the study who had ever practiced flashblood. The frequency of flashblood use could not be determined for all 6 users, as flashblood was only practiced when the users could not find heroin. All are current heroin injection users, and all also use non-injection heroin. All of the flashblood practitioners shared needles, shared injection equipment, have taken someone else’s syringe and leave syringes in a common location.

### Knowledge of risks posed by drug use and using needles

Knowledge and awareness about the risks posed by using and sharing syringes is crucial to HIV prevention (see Table 4). Approximately one third (32.3%) of the study population did not know about any kind of health risk posed by sharing syringes. While two-thirds (64.6%) was aware that HIV was a risk posed by sharing needles, fewer knew that sharing syringes could lead to hepatitis C (26.2%). Not knowing about the risks of using and sharing syringes was associated with more than 4 times the odds of injecting heroin compared to those who were aware of risks. More than half the study population was not aware of the general health problems associated with substance use (see Table 4).

**Table 4:** Selected unadjusted and adjusted associations between awareness of risks posed by using and sharing needles and injection drug use.

**Awareness of risks posed by sharing needles**	**PWID (Heroin)(n = 65)**	**Unadjusted odds ratio of injection drug use**	**Adjusted odds ratio of injection drug use**
Don’t know about risks	21/65 (32.3%)	6.87 (3.94–12.32)	4.55 (2.48–8.30)
HIV	42/65 (64.6%)	6.41(3.70–11.36)	-
Hepatitis C	17/65 (26.2%)	6.24 (3.13–12.32)	2.84 (1.32–6.18)
TB	10/65 (15.4%)	13.18 (4.39–44.21)	3.87 (1.16–15.21)
Mental problems	3/65 (4.6%)	9.99 (1.63–76.99)	-
**Health problems caused by any sort of illicit drugs**	**Total population**	**Injection Drug Users**	**Unadjusted odds ratio of heroin injection**
Don’t know	292 (60.8%)	34/65 (52.3%)	1.50 (0.88–2.53)
Tuberculosis	99 (20.6%)	12/65 (18.5%)	0.85 (0.42–1.62)
Loss of memory	78 (16.3%)	13/65 (20.0%)	1.35 (0.67–2.55)
Dehydration	42 (8.8%)	12/65 (18.5)	2.91 (1.36–5.90)
Loss of appetite	20 (4.2%)	3/65 (4.6%)	1.13 (0.26–3.50)

After adjusting for knowledge of other diseases, the adjusted multivariate model demonstrated that “not knowing” and knowledge of TB were the two main knowledge factors associated with heroin injection. The odds of injection among those who were not aware of any problems (“not knowing”) was 10.4 times (95% CI: 5.88–18.97) that of those who do know.

### Strongest factors associated with heroin injection

A reduced multivariate model demonstrated that housing and co-habitation status were significantly associated with heroin injection. However, after adjustment, housing status was no longer significantly associated with heroin injection. The odds of heroin injection among those who do not live with a spouse are 2.25 times (95% CI: 1.05–5.44) those who do live with a spouse. These results reinforce that results from Table 2 where both PWID and non-PWID cite family problems and peer pressure as the main reason for starting substance use.

Further, as shown in Table 5, multivariate analysis also demonstrated that years of drug use, selling items for drugs, trading sex for drugs and being arrested by law enforcers are strongly associated with injection. The odds of injection among those who have used drugs for 11–15 years and 16–20 are 1.09 times (95% CI: 1.00–1.18; 0.96–1.22) compared to those who have used drugs for 0–5 years. Those who reported selling items for drugs are 1.12 times as likely to inject heroin compared to those who have not. Similarly, those who have traded sex for drugs are 1.22 times significantly as likely to inject heroin compared to those who have not. The significance of these HIV-related risk behaviors in predicting heroin injection cannot be discounted.

**Table 5:** Reduced model of HIV related risk behaviors and injection drug use.

	Odds ratio of injection drug use	P-value
**Years since first use**		
0–5 years	1.00	-
6–10 years	0.96 (0.89–1.04)	0.36
11–15 years	1.09 (1.00–1.18)	0.06
16–20 years	1.09 (0.96–1.22)	0.17
**Ever sold items for drugs**		
Yes	1.12 (1.04–1.21)	<0.05
**Traded sex for drugs**		
Yes	1.22 (1.12–1.33)	<0.05
**Arrested by law enforcers**		
Yes	1.11 (1.00–1.22)	<0.05

## Discussion

To our knowledge, this is the first study to describe the epidemiology of substance use in general and substance injection in particular, and associated HIV related risk behaviors in Mwanza, Tanzania. Using a survey of 480 drug users, we found high numbers of heroin use, and HIV risk behaviors amongst current substance users in Mwanza. We also found marginal differences between PWID and non-PWID in terms of socioeconomic status. These results suggest that the population in these two sub-groups of drug users is similar and that their backgrounds are not confined to any one particular socioeconomic stratum.

Approximately one fifth of the study population had a history of heroin injection, and 13.5% were current heroin injectors. Very few people reported injection with other types of drugs besides heroin and combinations of smoking heroin and marijuana were also commonly reported. Given that the overwhelming majority of individuals with a history of injection are current injectors, this highlights the urgent need to establish harm-reduction oriented programs, including methadone maintenance, which are currently absent in Mwanza.

Marijuana and heroin are readily accessible in Mwanza. Study participants reported that approximately 0.5g of heroin costs 2000TSH (USD$1.10), and that 600g of marijuana cost 1000TSH (USD$0.55). Similar figures were reported a decade ago and show remarkably stable prices in the illicit drug market.[5] With such low prices, it is not surprising that heroin use has become relatively common in this population in northwestern Tanzania. Substance users who smoke heroin with marijuana may experience an increased probability of transitioning to injection. It is well-established that the median transition time from smoking to injection is 5 years, and for those under 25, it is only 2 years.[20] The availability of heroin, the low cost of heroin, and the high study participation rate within a short span of time, highlight that heroin use in Mwanza is a growing problem.[6]^,^[21]^,^[22]

Not surprisingly, we did not observe significant differences in the socioeconomic characteristic between PWID and non-PWID. Studies in Russia and other OECD countries have shown that high risk injection behaviors were not related to socioeconomic markers,[23]^,^[24] even among injectors who had experienced non-fatal overdoses.[25] Our data show that being single, having a primary level or lower secondary level of education, and being male were not significantly associated with heroin injection. This underscores the need for broad drug prevention efforts that are aimed at users from various socioeconomic backgrounds in Mwanza. [12] These results highlight the importance of home stability and family as protective factors against heroin injection.

Housing and cohabitation status were found to be the most significantly associated with injection drug use in our multivariate model. PWID are more likely to rent houses or rooms than to own homes. We also found that PWID are more likely to not have a spouse or co-habiting partner compared to non-PWID. These results suggest that both housing and marital status are closely associated with injection. Other heroin injection and HIV risk studies conducted outside of sub-Saharan Africa [26]^,^[27] have also shown that housing status and residence are key factors in increased HIV risk and injection. Lack of stable housing such as renting a room in a guesthouse was shown to be independently associated with several HIV risk behaviors. Our results suggest for strengthening family ties through treatment with methadone. One of the first signs of recovery in the Dar es Salaam methadone clinics is when the patient re-connects with family and is accepted back into the family.[28]

Deciphering the start of the heroin epidemic among our study population is difficult. The type of drug that was first used was not specified. Among our study participants, the mean length of drug use is 7.9 years. The most common year that people began to use drugs was in 2006, implying a recent uptick in heroin imports in the region. This is also consistent with UNODC reports that cite an increase in heroin seizures over the past decade, which also imply that there could be many more undetected shipments.[29] Heroin use is not a recent problem, but one that has remained invisible for many years in the region. Similar to what has been shown in other PWID studies[20], our data show that the longer the length of drug use, the greater the probability that the individual will transition to injection. If action is not taken to curb the transition from heroin smoking to injection, heroin use could continue to impact the HIV epidemic in Tanzania. Such inaction is evident in Russia where 75% of all HIV cases occur among PWID.[30] There remains an urgent need for action.

Risk behaviors that are significantly associated with heroin injection include: selling items for drugs, trading sex, and being arrested. These are known risk behaviors that are associated with an increased risk of heroin injection and HIV, as shown in previous studies in Dar es Salaam.[5]^,^[7] Among our study population, age of sexual debut, use of drugs during sex, condom use and number of sex partners were not found to be significantly associated with heroin injection. Reported frequent condom use during vaginal sex was also very low. An overwhelming majority of PWID and non-PWID alike began using drugs because of peer pressure. Importantly, in our study population, some started having sex at age 11. This is concerning as it has been found in rural Tanzania that HIV was more prevalent among 15–19 year olds who had never been to school or left school before year 5.[31] However, adolescent sexual health program interventions were found to not be significantly effective in the long-term to reduce HIV or STI prevalence.[32,33] With the early sexual debut of many of these individuals combined with previous findings in Tanzania, our results emphasize the need for interventions that address broader sexual norms and behaviors that should also include a component of substance use education.

Our data show that alcohol risk behaviors are not significantly associated with heroin injection. While it is known that alcohol consumption is implicated in the spread of HIV/AIDS,[8] our results show that daily binge drinkers are significantly less likely to engage in heroin injection compared to those who do not binge drink at all. Although not associated with injection, alcohol use in Tanzania is a growing problem in itself and was present in this study population (Table 3). [18]^,^ [34]^,^ [35]

Needle-related risk behaviors were widely reported among PWID. More than half the PWID leave syringes in common locations and half the PWID shared injection equipment. The general availability of syringes—which consists of a 2ml syringe and 23 gauge needle and is sold in a pack[5]—facilitates needle-related risk behaviors. Albeit this decreases the probability of HIV transmission among PWID, our results are still concerning. Taken with the fact that PWID are twice as likely to have sex with more than 5 partners, our data suggest that heroin injection in Mwanza is a serious issue that could result in further HIV spread.

Despite the widespread sharing of injection equipment, cleaning injection equipment was widely reported among PWID. The most commonly used cleaning agent is cold water, which is ineffective to sterilize the syringe. Ideally, PWID should clean their needles with bleach adequately to kill viruses,[19] which is currently only being practiced by 29.2% of the PWID in our study population. Community-based outreach organizations should continue to encourage cleaning with bleach in addition to reducing multi-person use of needles.

We investigated the practice of flashblood, which was first described in Dar es Salaam.[7]^,^[11]^,^[22] We found that although flashblood was not a significant concern among our study population, it is worrisome that the practice was not unheard of and had been practiced by 6 PWID. Flashblood was only used when circumstances were dire; i.e., when both money and heroin was unavailable. This practice has not been reported anywhere else except for East Africa.[36] Flashblood cannot be ignored, as the injection of blood obviates *all* HIV prevention measures to date.

In Mwanza, awareness about health risks posed by using and sharing needles is poor. A third of PWID were not able to list any health risks posed by using and sharing needles. Previous prevention efforts conducted in the Mbeya region of Tanzania have shown that information, education and communication about HIV is integral to the reduction of HIV prevalence.[37] However, information dissemination is only one part of the structural approach to HIV prevention; political support, involvement, institutional participation and surveillance are key features of successful programs for behavioral change in East Africa.[37]^,^[38] In agreement with these findings, our data suggests that knowledge alone is not enough as a prevention tool. For example, those who are aware about HIV, hepatitis C and TB are actually more likely to engage in heroin injection than those who are not. This suggests that it is not adequate to only raise awareness about diseases as a method of HIV prevention. In addition to knowledge, individuals must be provided the tools to prevent HIV–needle and syringe programs (NSP), methadone maintenance, etc. Although methadone is available in Dar es Salaam, it is currently not available in the northwestern region of Tanzania or any neighboring countries. With the presence of heroin use in Mwanza, it is likely that heroin has already moved into neighboring regions–namely Western Kenya, Uganda, and Rwanda. These countries must begin evaluating the possibility of heroin use in their regions.

### Contribution of study

The results of this study should be used to catalyze the implementation of methadone clinics as well as other forms of harm reduction programs that provide access to sterile injection equipment in Mwanza and cities beyond Dar es Salaam and Zanzibar. Our results have highlighted that there is a sizable population of heroin drug users in Mwanza, and that many PWID are engaging in high-risk behaviors. However, we must not exclude the non-PWID as well. Given the large number of non-PWID who use heroin in our study sample, there is a high chance that non-PWID will make the transition to injection later on in life. Research has shown that non-PWID exposed to treatment such as methadone maintenance and rehabilitation are less likely to initiate injection.[39]

### Study limitations

These results should be interpreted in light of the following limitations. Firstly, although great care was taken to ensure that we had a sizable and diverse sample of drug users, obtaining a random representative sample of hidden population is extremely difficult. We were also not able to recruit many female drug users, which may have skewed results. The nature of our study is also heavily reliant on self-report, which may have resulted in underreporting of risky behaviors that are not socially acceptable. However, self-report was and is still the main measure of risky behavior.[40] Further, our data are cross-sectional in nature, thus it is not possible to extrapolate the data to make causal inferences. Our data did not plot the chronological transition of users from using non-injection to injection heroin. This would have allowed us to find a suitable intervention point among drug users.

## Conclusion

The results of this study highlight that heroin use is a significant problem in a major city outside Dar es Salaam and Zanzibar, suggesting that this urban center has now become a consumer hub of heroin, and may be a transit hub for heroin to other parts of East Africa. We find that housing status and living arrangements are the strongest predictors of heroin injection, and that many users began using drugs due to peer pressure, underscoring the fact that family stability and home life should be taken into consideration in HIV and drug prevention efforts in Tanzania. Many PWID were also reported to engage in needle sharing and reported improper methods of cleaning needles. A significant proportion of the study population exhibited a lack of awareness of the risks involved in needle sharing and drug use. However, even among those who are knowledgeable about risks, the odds of heroin injection are high. This suggests that HIV prevention must extend beyond education campaigns, such as ensuring that students stay in schools, engaging in productive after school activities and encouraging family counseling. Failing to recognize heroin injection in Mwanza may derail the work that has been done to address the HIV epidemic in Tanzania. Furthermore, the high prevalence of heroin use in Mwanza should sound the public health alarm in neighboring countries. If heroin use is active in Mwanza, it is likely crossing Lake Victoria to Uganda and western Kenya, as well as continuing on the road to Kigali, Rwanda. The prevalence of heroin in Mwanza needs a broad response.

## Supporting Information

S1 Questionnaire Mwanza Risk Behavior Assessment Questionnaire. (PDF)
