# Supplementary material for: Epidemiology of Drug Use and HIV-Related Risk Behaviors among People Who Inject Drugs in Mwanza, Tanzania
Source: PLoS One. 2015 Dec 23;10(12):e0145578. doi: 10.1371/journal.pone.0145578 (PMC4689416; doi:10.1371/journal.pone.0145578)
Supplement: S1 Questionnaire — (PDF) [file pone.0145578.s001.pdf]

Section 1: Socio-Demographic Information

This first section is general information about yourself

	Questions and Filters	Coding Categories		SKIP
101	Write interviewer's name<br><br>____________________	Interviewer Initials<br><br>[____|____|____]		
102	Write date of interview	[___|___] [___|___|___] [___|___|___|___]<br>DAY MONTH YEAR		
103	Can you tell me your date of birth?<br><br>***Write 99 in DAY, 999 in MONTH, 9999 in YEAR if DON'T KNOW***	[___|___] [___|___|___] [___|___|___|___]<br>DAY MONTH YEAR		
104	Gender	MALE<br>FEMALE	1<br>2	
105	What is the highest level of education you have attained?	NEVER WENT TO SCHOOL<br>INCOMPLETE PRIMARY<br>COMPLETED PRIMARY (std 7)<br>SECONDARY (FORM ONE-FOUR)<br>SECONDARY (FORM FIVE-SIX)<br>INCOMPLETE POST SECONDARY (UNIVERSITY OR COLLEGE)<br>COMPLETED POST-SECONDARY (UNIVERSITY OR COLLEGE)<br>DON'T KNOW/REFUSE TO ANSWER	1<br>2<br>3<br>4<br>5<br>6<br>7<br>9	
106	What is your current marital status	MARRIED OR LIVING AS MARRIED (ONLY 1 HUSBAND/WIFE)<br>MARRIED OR LIVING AS MARRIED (MORE THAN 1 HUSBAND/WIFE)<br>WIDOWED<br>SEPARATED/DIVORCED<br>SINGLE (NEVER MARRIED)<br>DON'T KNOW/REFUSE TO ANSWER	1<br>2<br>3<br>4<br>5<br>9	
107	Do you currently live with your husband/wife or a sexual partner?	NO<br>YES	1<br>2	**109**
108	How long have you been living with this husband/wife or sexual partner?<br>***Write number of months and/or years.***	NUMBER OF MONTHS [____|____]<br>***(Write 00 for MONTHS if less than 1 month)***<br>NUMBER OF YEARS [____|____]<br>***(Write 00 for YEARS less than 1 year)***		
109	Living Arrangement	OWN HOUSE<br>RENTED HOUSE<br>RENTING ROOM IN A GUESTHOUSE<br>FREE ROOM IN A GUESTHOUSE<br>RENTING ROOM ELSEWHERE	1<br>2<br>3<br>4<br>5	

		FREE ROOM AT FRIENDS OR RELATIVE'S HOUSE<br>OTHER<br>SPECIFY:________________________________<br>DON'T KNOW/REFUSE TO ANSWER	6<br>7<br><br>9	
110	What is your main source of income?	JOB<br>SPOUSE/SEX PARTNER<br>FAMILY/FRIENDS<br>OTHER<br>SPECIFY:________________________________<br>DON'T KNOW/REFUSE TO ANSWER	1<br>2<br>3<br>4<br><br>9	
111	On average, how much do you earn each month from your main source of income? (***Probe so he/she gives you accurate answer. Request him/her to think about all earnings she gets from his/her main job***)	Tshillings [____|____|____|____|____|____]<br>***(Write 999999 if DON'T KNOW)***		

Section 2: Drug Use Risk Behaviors

Read: *This part of the survey is regarding drug use behaviors.. If you do not want to answer any of the questions, you do not have to.*

	Questions and Filters	Coding Categories		SKIP
201	What drugs have you ever used?<br>(**Select all that apply**)	Cannibinoids – resin – hashish<br>Cannibinoids – marijuana<br>Sniffing petrol/solvent<br>Opoids (heroin/cocaine)<br>Pharmaceuticals<br>Amphetamines<br>Other:<br>_______________<br>DON'T KNOW/REFUSE TO ANSWER	1<br>2<br>3<br>4<br>5<br>6<br>7<br><br>9	
202	What drugs have you used in the past 12 months?<br>(**Select all that apply**)	Cannibinoids – resin – hashish<br>Cannibinoids – marijuana<br>Sniffing petrol/solvent<br>Opoids (heroin/cocaine)<br>Pharmaceuticals<br>Amphetamines<br>Other:<br>_______________<br>DON'T KNOW/REFUSE TO ANSWER	1<br>2<br>3<br>4<br>5<br>6<br>7<br><br>9	

203	What drugs are you <u>currently</u> using? (**Select all that apply**)	Cannibinoids – resin – hashish<br>Cannibinoids – marijuana<br>Sniffing petrol/solvent<br>Opoids (heroin/cocaine)<br>Pharmaceuticals<br>Amphetamines<br>Other:<br>______________<br>DON'T KNOW/REFUSE TO ANSWER	1<br>2<br>3<br>4<br>5<br>6<br>7<br><br>9	
204	When was the last time you used any of the drugs you have mentioned?<br><br>***Write 99 in DAY or 999 in MONTH if DON'T KNOW***<br>***Write 9999 in YEAR if DON'T KNOW***	[___|___] [___|___|___] [___|___|___|___]<br>DAY MONTH YEAR		
205	When did you start using drugs?<br>***Write 99 in DAY or 999 in MONTH if DON'T KNOW***<br>***Write 9999 in YEAR if DON'T KNOW***	[___|___] [___|___|___] [___|___|___|___]<br>DAY MONTH YEAR		
206	<u>On average</u>, how many days a week do you use drugs of any kind?	NUMBER OF DAYS PER WEEK [___]<br>***(Write 0 if NONE***<br>***9 if UNKNOWN)***		
207	<u>On average</u>, how many times a day do you use drugs?	NUMBER OF TIMES A DAY [___|___]<br>***(Write 00 if NONE)***<br>***(Write 99 if UNKNOWN***		
208	Have you <u>ever</u> injected any of the following drugs? (**Select all that apply**)	Heroin<br>Cocaine<br>Methamphetamine<br>Other<br>______________<br>NONE<br>DON'T KNOW/REFUSE TO ANSWER	1<br>2<br>3<br>4<br><br>5<br>9	 <br><br><br><br>**210**
209	Are you <u>currently</u> injecting any of the following drugs? (**Select all that apply**)	Heroin<br>Cocaine<br>Methamphetamine<br>Other<br>______________<br>NONE<br>DON'T KNOW/REFUSE TO ANSWER	1<br>2<br>3<br>4<br><br>5<br>9	
210	Are you <u>currently</u> using any of the following non-injection drugs? (**Select all that apply**)	Cannibinoids – resin – hashish<br>Cannibinoids – marijuana<br>Sniffing petrol/solvent<br>Opoids (heroin/cocaine)<br>Pharmaceuticals<br>Other:	1<br>2<br>3<br>4<br>5<br>6	

		___________<br>NONE<br>DON'T KNOW/REFUSE TO ANSWER	 7<br>9	
211	*If you are a current injection drug user*, have you ever shared needles/syringes with other users?	NO<br>YES<br>DON'T KNOW/REFUSE TO ANSWER	1<br>2<br>9	**215**<br><br>**215**
212	Do you leave syringes in common locations where other drug users may have access to them?	NO<br>YES<br>DON'T KNOW/REFUSE TO ANSWER	1<br>2<br>9	
213	Do you know if another drug user has taken your syringe and used it?	NO<br>YES<br>DON'T KNOW/REFUSE TO ANSWER	1<br>2<br>9	
214	Did you share other injection equipment (like cookers) with other users at last injection?	NO<br>YES<br>DON'T KNOW/REFUSE TO ANSWER	1<br>2<br>9	
215	Did you clean injection equipment such as needles and cookers?	NO<br>YES<br>DON'T KNOW/REFUSE TO ANSWER	1<br>2<br>9	**217**<br><br>**217**
216	Did you clean injection equipment such as needles and cookers?	SOAP AND WATER<br>ALCOHOL<br>BLEACH<br>BOILING WATER<br>OTHER WAYS<br>SPECIFY:______________<br>DON'T KNOW/REFUSE TO ANSWER	1<br>2<br>3<br>4<br>5<br><br>9	
217	Have you ever practiced "flashblood"?<br>(***Explain what flashblood is: Flashblood is the practice in which you inject yourself with the blood of another drug user who has just recently injected***)	NO<br>YES<br>DON'T KNOW/REFUSE TO ANSWER	1<br>2<br>9	**219**<br><br>**219**
218	If ***YES***, how many times do you practice flashblood a week?	[______________] TIMES<br>***(WRITE 999 FOR DON'T KNOW/REFUSE TO ANSWER)***		
219	Have you ever sold items for drugs?	NO<br>YES<br>If YES, please specify what items:<br>______________________<br>DON'T KNOW/REFUSE TO ANSWER	1<br>2<br><br><br>9	
220	Have you ever traded sex for drugs?	NO<br>YES<br>DON'T KNOW/REFUSE TO ANSWER	1<br>2<br>9	

221	Have you ever gotten in trouble with the law while doing drugs?	NO<br>YES<br>DON'T KNOW/REFUSE TO ANSWER	1<br>2<br>9	
222	Where do you get your drugs?	DEALER<br>FRIENDS<br>FAMILY<br>PHARMACY<br>OTHER:<br>___________________<br>DON'T KNOW/REFUSE TO ANSWER	1<br>2<br>3<br>4<br>5<br><br>9	
223	Is your husband/wife or sexual partner also a drug user?	NO<br>YES<br>DON'T KNOW/REFUSE TO ANSWER	1<br>2<br>9	
224	What was the main reason you started doing drugs?	CURIOSITY<br>PEER PRESSURE<br>FOR FUN<br>FAMILY PROBLEMS<br>RELATIONSHIP PROBLEMS<br>INFLUENCE FROM FAMILY<br>IMPROVE SEXUAL PERFORMANCE<br>OTHER: ____________________<br>DON"T KNOW/REFUSE TO ANSWER	1<br>2<br>3<br>4<br>5<br>6<br>7<br>8<br>9	
225	Can you mention health problems associated with using drugs? (**Write the problems mentioned. If don't know or refuse to answer select 9**)	________________________<br>________________________<br>DON'T KNOW/REFUSE TO ANSWER	9	
226	Can you mention health problems associated with sharing needles/syringes with other users? (**Write the problems mentioned. If don't know or refuse to answer select 9**)	________________________<br>________________________<br>DON'T KNOW/REFUSE TO ANSWER	9	

Section 3: Sex Risk Behaviors

	Questions and Filters	Coding Categories		SKIP
301	How old were you at the first time you had sexual intercourse?	AGE [____|____]<br>***(Write 99 if DON'T KNOW)***		
302	The <u>last time</u> you had sex, did you use a condom? This could be either a male or female condom.	**NO YES DON'T KNOW**<br>MALE CONDOM 1 2 9<br>FEMALE CONDOM 1 2 9		
303	The <u>last time</u> you had sex, did you give/receive sex for drugs or money? (***Here***, "***drugs" means illicit drugs)***	NO<br>YES<br>DON'T KNOW/REFUSE TO ANSWER	1<br>2<br>9	
304	Do you <u>regularly</u> use drugs while having sexual intercourse? (***regularly*** = ***more than half the time***)	NO<br>YES<br>DON'T KNOW/REFUSE TO ANSWER	1<br>2<br>9	**306**<br><br>**306**
305	If <u>YES</u>, what kind of drugs do you use?	Cannibinoids – resin – hashish<br>Cannibinoids – marijuana<br>Sniffing petrol/solvent<br>Opoids (heroin/cocaine)<br>Opoids pharmaceuticals<br>Amphetamines<br>Other:<br>_______________<br>DON'T KNOW/REFUSE TO ANSWER	1<br>2<br>3<br>4<br>5<br>6<br>7<br><br>9	
306	Thinking about the man/woman who you last had sex with, what is your relationship with this man/woman?<br><br>***Record "OTHER REGULAR PARTNER" " if they have relationship for at least 1 year***	HUSBAND/WIFE<br>CO-HABITING PARTNER<br>OTHER REGULAR<br>CASUAL AQUAINTANCE<br>OTHER<br>Specify:________________<br>DON'T KNOW/REFUSE TO ANSWER	1<br>2<br>3<br>4<br>5<br><br>9	
307	In the <u>last 30 days,</u> how many different partners have you had sex with in total?	NUMBER OF PARTNERS [____|____]<br>***(Write 99 if DO NOT REMEMBER/DON'T KNOW/REFUSE TO ANSWER; write 00 if HAD NO SEX)***		

308	In the last 30 days, how often did you use condoms when having vaginal sex?	Not had sex in the past 30 days<br>All the time<br>Most of the time<br>Some of the time<br>None of the time<br>DON'T KNOW/REFUSE TO ANSWER	1<br>2<br>3<br>4<br>5<br>9	
309	During the last 6 months, how many different partners have you had sex with in total?	NUMBER OF PARTNERS [____|____|____]<br>***(Write 999 if DO NOT REMEMBER***<br>***Write 000 for NONE or HAD NO SEX)***		
310	In the last 6 months, how often did you use condoms when having vaginal sex?	Not had sex in the past 6 months<br>All the time<br>Most of the time<br>Some of the time<br>None of the time<br>DON'T KNOW/REFUSE TO ANSWER	1<br>2<br>3<br>4<br>5<br>9	
311	In the last 6 months, how often did you use condoms when having anal sex?	Not had sex in the past 6 months<br>All the time<br>Most of the time<br>Some of the time<br>None of the time<br>DON'T KNOW/REFUSE TO ANSWER	1<br>2<br>3<br>4<br>5<br>9	
312	How would you describe your sexual orientation?	STRAIGHT<br>HOMOSEXUAL<br>BISEXUAL<br>OTHER (PLEASE SPECIFY):<br>________<br>DON'T KNOW/REFUSE TO ANSWER	1<br>2<br>3<br>4<br><br>9	
313	In the last 30 days, did you have anal sex?	NO<br>YES<br>DON'T KNOW/REFUSE TO ANSWER	1<br>2<br>9	
314	In the last 30 days, how often did you use condoms when having anal sex?	Not had sex in the past 30 days<br>All the time<br>Most of the time<br>Some of the time<br>None of the time<br>DON'T KNOW/REFUSE TO ANSWER	1<br>2<br>3<br>4<br>5<br>9	

Part 4: Alcohol Risk Behaviors

Read: This part of the survey is regarding alcohol consumption. If you do not want to answer any questions, you do not have to.

	Question and filters	Coding categories	Skip
401	Have you ever drunk an alcohol-containing beverage? For example beer, wine, local brew, or other alcoholic beverage?	NO 1<br>YES 2<br>DON'T KNOW/REFUSE TO ANSWER 9	**END**
402	In the past one year (12 months), have you drunk an alcohol-containing beverage? For example beer, wine, local brew, or other alcoholic beverage?	NO 1<br>YES 2<br>DON'T KNOW/REFUSE TO ANSWER 9	**END**<br><br>**END**
403	What alcohol-containing beverage do you drink usually?<br>BOTTLED BEER<br>WINE<br>SPIRIT/LIQUOR<br>LOCAL BEER/ALCOHOLIC DRINK<br>OTHER	NO YES<br>……………………………………1 2<br>……………………………………1 2<br>……………………………………1 2<br>……………………………………1 2<br>(Specify:________________________)	
404	How often do you have a drink containing alcohol?	NEVER 1<br>MONTHLY OR LESS 2<br>2-4 TIMES A MONTH 3<br>2-3 TIMES A WEEK 4<br>4 OR MORE TIMES A WEEK 5<br>DON'T KNOW 9	
405	How often do you have six or more drinks on one occasion? (***Probe***)	NEVER 1<br>LESS THAN MONTHLY 2<br>MONTHLY 3<br>WEEKLY 4<br>DAILY OR ALMOST DAILY 5<br>DON'T KNOW 9	
406	How often during the last year have you found that you were not able to stop drinking once you had started? (***Probe***)	NEVER 1<br>LESS THAN MONTHLY 2<br>MONTHLY 3<br>WEEKLY 4<br>DAILY OR ALMOST DAILY 5<br>DON'T KNOW 9	
